# *Campylobacter hepaticus* Transcriptomics Identified Genes Involved in Spotty Liver Disease (SLD) Pathogenesis

**DOI:** 10.3390/pathogens14101048

**Published:** 2025-10-17

**Authors:** Varsha Bommineni, Lekshmi K. Edison, Chaitanya Gottapu, Gary D. Butcher, Subhashinie Kariyawasam

**Affiliations:** 1 Department of Comparative Diagnostics and Population Medicine, University of Florida, Gainesville, FL 32608, USA; vbommineni@ufl.edu (V.B.); edison.le@ufl.edu (L.K.E.); cgottapu@ufl.edu (C.G.); 2 Department of Large Animal Clinical Sciences, University of Florida, Gainesville, FL 32608, USA; butcher@ufl.edu

**Keywords:** *Campylobacter hepaticus*, layer chickens, Spotty Liver Disease, transcriptomics, bile stress, intracellular persistence, poultry pathogen, virulence mechanisms

## Abstract

*Campylobacter hepaticus* is the etiological agent of Spotty Liver Disease (SLD), a newly emerging bacterial disease of laying hens resulting in significant mortality and production losses primarily in free-range systems. Although its economic impact continues to grow, the molecular basis of *C. hepaticus* pathogenesis remains poorly understood. In this study, we conducted transcriptomic profiling of *C. hepaticus* in three host-relevant conditions, exposure to chicken bile, infection of a chicken liver hepatocellular carcinoma (LMH) cell line, and isolation from liver lesions of naturally infected chickens. Through RNA-seq analysis, we found unique gene expression signatures in each environment. In the bile, *C. hepaticus* exhibited differential expression of 412 genes, with upregulation of genes related to motility, cell envelope remodeling, glycosylation, nitrate respiration, and multidrug efflux systems, indicating a stress-adaptive, metabolically active lifestyle. In LMH, on the other hand, 125 genes were differentially expressed, primarily reflecting downregulation of motility, oxidative stress response, chaperones, and core metabolic processes, suggesting that these cells adopt a less active, intracellular dormant lifestyle. Transcriptomic analysis of *C. hepaticus* isolated from the liver identified 26 differentially expressed genes, featuring selective upregulation of genes associated with nitrate respiration, sulfur metabolism, and pyridoxal 5’ phosphate homeostasis, alongside downregulation of the major outer membrane porin (*momp*), stress response chaperones (*dnaK*, *groL*), and genes involved in oxidative stress defense and energy production. Furthermore, the immune evasion-related gene *cmeA* and a glycosyltransferase gene were found to be highly upregulated. This study presents the first in-depth transcriptomic exploration of *C. hepaticus* in multiple host relevant niches. Our findings reveal niche-specific gene expression profiles and highlight metabolic and structural adaptations that enable *C. hepaticus* to survive during bile exposure, persist within host cells, and contribute to liver pathology. These insights provide a basis for identifying novel virulence determinants and may inform the development of targeted interventions, including vaccines or antimicrobial therapy, to control SLD in commercial poultry operations.

## 1. Introduction

Spotty Liver Disease (SLD) is an infectious disease of poultry that causes multifocal whitish-gray liver lesions and is associated with high mortality and egg-production losses [1,2]. First described more than half a century ago, an SLD-like disease was sporadically reported in laying hens in the United States and Europe (often described as a “vibrionic hepatitis” or similar syndrome) prior to falling largely out of attention for many decades [3]. However, in recent years, SLD has re-emerged as an economically important disease in egg production systems worldwide. Outbreaks can lead to flock mortalities approaching 10–15% and egg production losses of up to 35%. The impact is especially severe in free-range and cage-free layer operations, where SLD now occurs with a higher frequency. Field reports and surveys strongly suggest that flocks provided with outdoor access or floor housing are at increased risk for SLD compared to caged layer flocks [4]. Indeed, the industry trend toward free-range management has coincided with the re-emergence of SLD outbreaks in countries such as Australia, the United Kingdom, and the United States [3]. Thus, this disease represents a significant threat to the health and productivity of commercial layer chickens, underscoring the need for further research into its causes and control measures.

The causative agent of SLD was not determined until the 2010s. Early studies reported the presence of *Campylobacter*-like organisms within the livers of compromised chickens, but positive identification was elusive. It was only in 2015 that Crawshaw et al. isolated a novel thermophilic *Campylobacter* from diseased hens in the United Kingdom [5,6]. Shortly thereafter, Van et al. recovered and described a similar bacterium from layers suffering from SLD and officially designated it as *Campylobacter hepaticus* in 2016, in Australia [7,8]. These studies marked a major breakthrough in understanding the causative agent of SLD. Subsequent experimental infections confirmed that oral challenge of healthy hens with pure cultures of *C. hepaticus* produced characteristic liver lesions and disease, with re-isolation of the bacterium from the liver and bile [8,9]. Notably, *C. hepaticus* has extensive invasiveness in vitro, shows the capacity to enter and survive within chicken hepatocyte cell cultures, and develops disease within several days of introduction to susceptible hens [8]. By 2018, the organism had been recovered on several continents, including from Australian free-range flocks experiencing outbreaks and the first documented instances in the United States [10]. The establishment of *C. hepaticus* as the causative agent of SLD ended a long-running enigma and categorized SLD as a bacterial disease, thus enabling directed research into its pathogenesis and epidemiology.

Although *C. hepaticus* has been identified as the causative agent of SLD, its pathogenesis remains poorly understood. It is further complicated by its fastidious, slow-growing nature, requiring precise microaerobic conditions, narrow temperature ranges (growing at 37–42 °C but not at 25 °C), and prolonged incubation times of 3 to 7 days for visible colony formation [11]. Unlike *C. jejuni* or *C. coli*, which colonize poultry relatively harmlessly, *C. hepaticus* causes severe disease in layer chickens. Although it has been isolated from the liver, bile, intestines, caeca, and cloacal swabs of infected chickens, the specific mode of dissemination to the liver is unclear [12]. Histopathological analyses have revealed necrotic hepatitis with associated inflammatory cell infiltrates [1,13]. However, the bacterial determinants that trigger the necrotic hepatitis and associated inflammatory infiltrates observed in histopathological analyses are unclear.

Notably, *C. hepaticus* lacks many of the typical classical virulence and stress-response genes possessed by other species of *Campylobacter*, including the cytolethal distending toxin (CdtA/B/C) gene cluster and multiple iron acquisition systems, thus possibly relying on alternative strategies, such as metabolic adaptation or immune evasion, to establish infection [3,12]. Observational field data indicate that stressors associated with the flock, such as competition for nest space and delay in egg production, often occur before the outbreak and highlight the possible role of host factors in the pathogenesis of the disease [14]. Key questions remain about the processes by which *C. hepaticus* survives, transmits itself, and causes disease within its host. In addition, stress response mechanisms are critical for *Campylobacter* survival during host colonization and disease. In other *Campylobacter* species, systems such as oxidative stress defenses (e.g., superoxide dismutase, thioredoxin, and catalase), heat shock chaperones (e.g., GroEL, DnaK), and bile resistance mechanisms (e.g., the CmeABC efflux pump) play central roles in persistence and virulence under hostile host conditions [15,16,17,18]. Interestingly, *C. hepaticus* lacks several of these classical stress-response genes, suggesting that it may rely on alternative pathways such as metabolic remodeling, envelope modification, and immune evasion to withstand oxidative and bile stress in the chicken liver. Understanding these compensatory stress responses is essential to unravel the unique virulence markers of *C. hepaticus* and SLD pathogenesis.

Recent genomics and transcriptomics studies have begun to elucidate the biology of *C. hepaticus*. Its genome is notably smaller (approximately 1.5 Mb, G+C content of 28%) than the genomes of other species of *Campylobacter* [19,20], suggesting a characteristic diminution in genomic content adapted to the chicken liver environment [9]. Transcriptomic profiling has also provided insights into the survival of *C. hepaticus* in vivo. Van et al. demonstrated that, in comparison to in vitro culture, bacteria recovered from infected bile upregulated genes associated with stress response, nutrient acquisition, and metabolic pathways, such as glucose metabolism, hydrogen metabolism, and sialic acid modification [12]. These results imply that metabolic adaptation and stress tolerance are critical for *C. hepaticus* survival in the bile during infection. However, this study was restricted to bile vs. Brucella broth media, and bacterial gene expression in other environmental niches remains to be investigated.

To fill this gap, we performed RNA-sequencing of *C. hepaticus* in three host-relevant niches: bile, chicken liver hepatocellular carcinoma epithelial (LMH) cells, and infected liver tissue collected from SLD chickens. By comparing *C. hepaticus* gene expression in these host-relevant niches to that of bacteria grown in standard microaerophilic conditions in culture media (control), we aimed to identify niche-specific gene expression patterns that contribute to virulence, metabolism, and stress adaptation. This study provides the first in-depth transcriptomic map of *C. hepaticus* across infection-relevant environments and yields new insights into SLD pathogenesis and targets for the development of control measures.

## 2. Materials and Methods

### 2.1. Bacterial Strain and Culture Conditions

*Campylobacter hepaticus* type strain HV10 NCTC 13823^T^ (The National Collection of Type Cultures (NCTC), Porton Down, Salisbury, UK) was grown on Thermo Scientific Columbia Agar with 5% Sheep Blood (Thermo Fisher Scientific, Waltham, MA, USA) under microaerophilic conditions using Mitsubishi AnaeroPack-MicroAero gas generator (Thermo Fisher Scientific, Waltham, MA, USA) at 37 °C in an airtight chamber for 72 h to facilitate optimum growth of the bacteria [19,20,21]. Following incubation, well-isolated colonies were carefully picked and inoculated into Remel Brucella broth (Thermo Fisher Scientific, Lenexa, KS, USA). Cultures were incubated for two days at 37 °C under microaerophilic conditions. The bacterial suspensions were then centrifuged, and the resulting cell pellets were harvested for RNA extraction. Total RNA isolated from these bacterial pellets represented *C. hepaticus* grown in Brucella broth under standard microaerophilic laboratory culture conditions and served as the reference baseline for differential expression analysis against the three host-relevant niches (Bile, LMH, and liver) used in this study. Bacterial suspensions containing 1 × 10^8^ CFU/mL of *C. hepaticus* were used for in vitro inoculation experiments. 

The type strain HV10 (NCBI accession # CP065357.1) has been extensively characterized and its genome shows a very high similarity (>99.9%) to the genomes of *C. hepaticus* isolates from the U.S., such as UF2019SK1 (GenBank accession # CP065357.1), USA1 (GenBank accession # CP166729.1), USA5 (GenBank accession # CP166688.1), and USA52 (GenBank accession # CP063536.1) [3].

### 2.2. In Vitro Exposure of C. hepaticus to Chicken Bile

To mimic the biliary environment, *C. hepaticus* (1 × 10^8^ CFU/mL) was resuspended in sterile-filtered bile collected aseptically from the gallbladders of apparently healthy *C. hepaticus*-free commercial layer chickens during routine necropsy. The collected bile was immediately filtered through a 0.22 µm filter to remove debris and stored on ice until further use. Bacterial suspensions were incubated at 37 °C under microaerophilic conditions using Mitsubishi AnaeroPack-MicroAero gas generator (Thermo Fisher Scientific, Waltham, MA, USA) in a leak-proof container for 72 h. After incubation, the suspension was centrifuged to pellet both bacterial cells and bile components. The pellet was then stored in RNAlater (Thermo Fisher Scientific, Lenexa, KS, USA) for RNA stabilization, at −80 °C until total RNA extraction. Three independent bile samples (*n* = 3 biological replicates) were processed for transcriptomic analysis.

### 2.3. Chicken Liver Hepatocellular Carcinoma Epithelial Cells (LMH) and Infection

Chicken liver hepatocellular carcinoma epithelial cells (LMH; CRL-2117, Manassas, VA, USA) were cultured in Waymouth’s medium (Waltham, MA, USA) supplemented with 10% fetal bovine serum (FBS; Atlanta Biologicals, Flowery Branch, GA, USA) and maintained at 37 °C in a humidified atmosphere containing 5% CO_2_. For infection assays, 2 × 10^6^ LMH were seeded into T75 flasks (Corning Life Sciences, Tewksbury, MA, USA) pre-coated with 0.1% gelatin (Cell Biologics, Inc., Chicago, IL, USA) and allowed to grow until confluent monolayers were established [8]. To infect the cells, *C. hepaticus* from 72 h culture was washed with sterile phosphate-buffered saline (PBS), resuspended in the respective cell growth media, added at a multiplicity of infection (MOI) of approximately 100:1 bacteria-to-cell ratio per flask, and incubated for 12 h under the same culture conditions. After incubation, the infected monolayers were gently washed with sterile PBS, harvested by centrifugation, and the resulting pellets were stored in RNAlater (Thermo Fisher Scientific, Lenexa, KS, USA) at −80 °C until total RNA extraction. Three independent infection assays (*n* = 3 biological replicates) were conducted for transcriptomic analysis.

### 2.4. Liver Sample Collection from Spotty Liver Disease (SLD) Affected Chickens

Liver samples were collected from commercial layer chickens during a naturally occurring, confirmed SLD outbreak under the direction of a poultry extension veterinarian. All affected chickens were 31 weeks of age and at peak egg production. The flock experienced increased mortality, and necropsy of affected birds revealed characteristic white foci on the surfaces of the livers, consistent with SLD. In addition to clinical signs and necropsy findings, *C. hepaticus* was re-isolated from liver tissue samples and confirmed by culture, PCR, and 16S rRNA gene sequencing as previously detailed [7,10]. To further verify the isolated species, a *Campylobacter bilis*-specific glycerol kinase gene PCR was employed, ensuring that the isolated *Campylobacter* species was indeed *hepaticus* and not *bilis* [22]. Necropsy and laboratory testing confirmed that the affected chickens were free of other concurrent diseases.

Liver tissues containing white foci were collected during necropsy and immediately preserved in RNAlater (Thermo Fisher Scientific, Lenexa, KS, USA). Samples were stored at −80 °C until total RNA extraction was performed. For transcriptomic analysis, three independent liver samples (*n* = 3 biological replicates) were processed for RNA extraction. As samples were obtained from naturally deceased birds during a disease outbreak investigation, no experimental infection or euthanasia was performed, and therefore, ethical approval was not applicable.

### 2.5. RNA Extraction, rRNA Depletion, and mRNA Enrichment

Total RNA was extracted using the RiboPure RNA Purification Kit (Invitrogen, Waltham, MA, USA). In the case of LMH and liver tissue samples, bacterial RNA was selectively enriched with the MICROBEnrich Kit (Invitrogen, Waltham, MA, USA) to deplete host RNA. All samples were then subjected to rRNA depletion to enrich bacterial mRNA with the MICROBExpress Bacterial mRNA Enrichment Kit (Invitrogen, Waltham, MA, USA). All processes were performed following the manufacturer’s instructions. RNA quality and quantity were determined at each step using a Qubit 4.0 Fluorometer (Thermo Fisher Scientific, Wilmington, DE, USA).

### 2.6. Transcriptome Library Preparation, Sequencing, and Differential Gene Expression Analysis

Approximately 100 ng of enriched bacterial mRNA from each sample was used for cDNA library construction using the TruSeq Stranded mRNA Library Prep Kit (Illumina Inc., San Diego, CA, USA). The mRNA was enzymatically fragmented and used for synthesis of first- and second-strand cDNA. The resulting double-stranded cDNA fragments were adenylated at the 3’ ends and ligated with TruSeq RNA Combinatorial Dual Index Adapters (Illumina Inc., San Diego, CA, USA), followed by PCR amplification to enrich the libraries. The indexed cDNA libraries were multiplexed and clustered across two lanes of a flow cell, and sequencing was performed using the Illumina NovaSeq X Series (Illumina Inc., San Diego, CA, USA) platform. Raw RNA sequencing data were processed using CLC Genomics Workbench V.25.0.2 (Qiagen, Redwood City, CA, USA). Adapter sequences and low-quality reads (Phred score < 30) were removed. Cleaned reads were mapped to the *C. hepaticus* UF2019SK1 reference genome (NCBI Genome Assembly: ASM1577487v1), which was identified and deposited by our laboratory. For validation, mapping was also performed against *C. hepaticus* strain HV10 (NCBI Genome Assembly: ASM168747v2) reference genomes, yielding comparable results. Differential gene expression (DGE) analysis was conducted to compare transcriptomic profiles across bile-exposed, LMH-infected, and liver-derived samples. Genes were considered differentially expressed, following the criteria outlined in the previous studies, when they exhibited a *p*-value < 0.01, a false discovery rate (FDR) < 0.05, and a fold change (FC) > 2 [23,24]. All experimental conditions, including bile exposure, LMH infection, and liver-derived samples, were tested in three independent biological replicates each, providing robust and reproducible transcriptomic data. *C. hepaticus* grown in Brucella broth under standard microaerophilic conditions served as the control.

### 2.7. RT-qPCR Validation of RNA-Seq Results

Total RNA was extracted using the RiboPure RNA Purification Kit (Invitrogen, Waltham, MA, USA). For each sample, 200 ng of RNA from the control bacterial culture and from each treatment group (bile-exposed bacteria, LMH infection, and SLD-infected liver) was reverse transcribed with the iScript cDNA Synthesis Kit (Bio-Rad Laboratories Inc., Hercules, CA, USA). To validate the RNA-seq expression patterns, six *C. hepaticus* genes (*napH*, *groL*, *flaA*, *waaC*, *flgI*, and *cheV*) were quantified by reverse transcription–quantitative PCR (RT-qPCR). Amplification was performed using SsoAdvanced Universal SYBR^®^ Green Supermix (Bio-Rad) with gene-specific primers (listed in Supplementary Table S1) on a QuantStudio™ 5 Real-Time PCR System (Applied Biosystems, Carlsbad, CA, USA). The cycling conditions consisted of an initial denaturation at 95 °C for 3 min, followed by 40 cycles of 95 °C for 15 s and 60 °C for 30 s. Gene expression levels were normalized using the reference gene *rpoB*.

### 2.8. Data Availability

The transcriptomic profile data (raw and processed) described in this study were deposited in the Gene Expression Omnibus (GEO) database in NCBI under the accession number GSE305414.

## 3. Results

### 3.1. Overall Transcriptomic Response of C. hepaticus Across Different Host-Associated Environments

**Figure 1:**
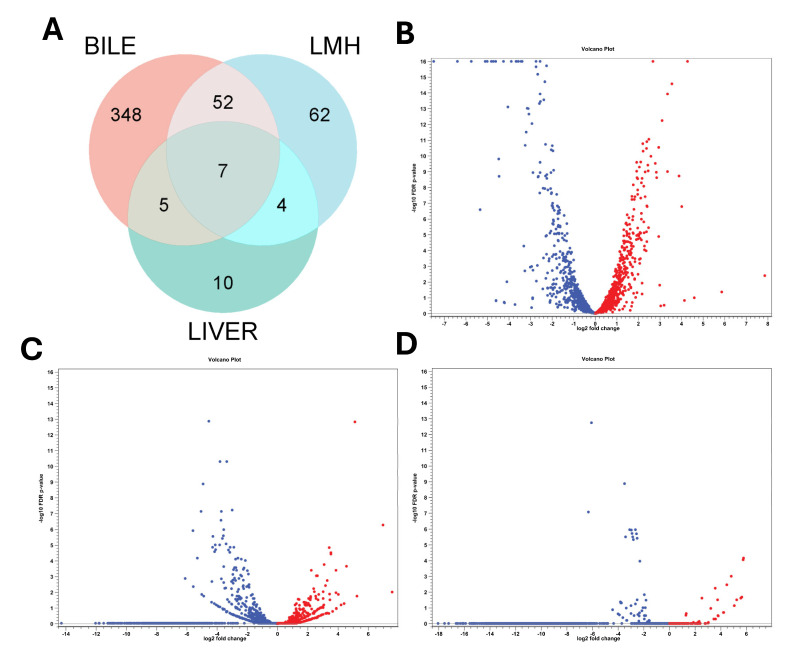
Overall transcriptomic response of *C. hepaticus* in bile, LMH, and liver. (**A**) Venn diagram depicting the overlap and unique differentially expressed genes (DEGs) in *C. hepaticus* under all three conditions. Volcano plots depicting the differential transcriptomic profile of *C. hepaticus* in (**B**) bile exposure, (**C**) LMH infection, and (**D**) infected liver. Each plot shows the relationship between the magnitude of gene expression change (X-axis = log_2_ fold change) and statistical significance (Y-axis = –log_10_ FDR *p*-value). Each dot represents a single gene; red indicates upregulation, and blue indicates downregulation.

Transcript reads obtained under all three conditions (in vitro bile, LMH infection, and SLD infected liver samples) mapped with high rates to the *C. hepaticus* reference genomes, UF2019SK1 reference genome (NCBI Genome Assembly: ASM1577487v1), and HV10 (NCBI Genome Assembly: ASM168747v2). Mapping to UF2019SK1 provided strong transcriptome coverage, between 98.7 and 99.8% for bile samples, 99.3–99.6% for LMH samples, and 99.3% for liver samples. Mapping rates to HV10 ranged between 98.76 and 99.8% for bile samples, 99.3 and 99.6% for LMH samples, and 99.2 and 99.3% for liver samples. RNA Integrity Number (RIN) values of 6 to 7 for all samples confirmed the quality and integrity of the RNA. Differential expression analysis identified unique transcriptional responses. Supplementary File S1 provides detailed, unfiltered differential gene expression profiles for each condition using *C. hepaticus* UF2019SK1 as reference genome. Supplementary File S2 provides detailed, unfiltered differential gene expression profiles for each condition using *C. hepaticus* HV10 as reference genome. After applying the filtration parameters, 412 differentially expressed genes (DEGs) were identified under the bile exposure condition, with 189 upregulated and 223 downregulated. In LMH, 125 DEGs were found, of which 29 were upregulated and 96 were downregulated, suggesting a shift toward a less metabolically active intracellular state. In liver tissue, 26 DEGs were found, including nine upregulated and 17 downregulated, indicating ongoing adaptation during infection. Of these, only seven genes were consistently differentially expressed across all three niches, all of which were downregulated. These included *flaA*, *groEL*, *rrf1*, *rrf2*, *rrf3*, *ssrA*, and a DUF2910 family protein I5Q61_RS04730 (Figure 1A). These results underscore the dynamic transcriptional reprogramming of *C. hepaticus* to accommodate various host environments during infection. Volcano plots showing the differential gene expression patterns are presented in Figure 1B–D, while heat maps of gene expression profiles are provided in Supplementary Figures S1–S3.

### 3.2. Transcriptomic Response of C. hepaticus to Bile Exposure

**Figure 2:**
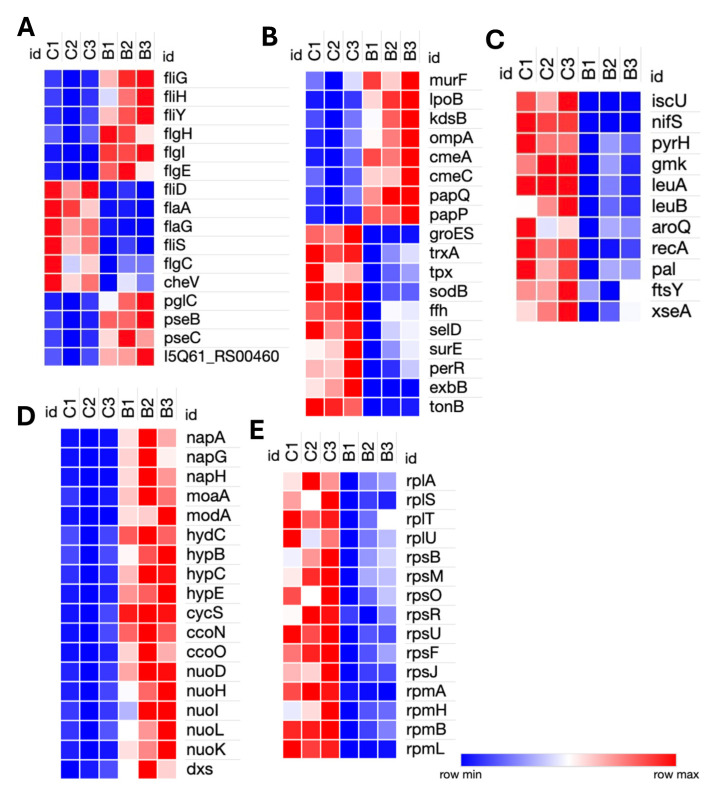
Heatmap of differentially expressed genes in *C. hepaticus* in bile-treated vs. control (bacteria grown in Brucella broth under standard microaerophilic culture) conditions. (**A**) Genes involved in motility, chemotaxis, surface structure modification, and glycosylation. (**B**) Genes related to peptidoglycan and lipooligosaccharides (LOS) biosynthesis, outer membrane proteins, multidrug efflux pumps, ABC transporters, and stress response chaperones. (**C**) Genes associated with nitrate respiration, molybdenum cofactor biosynthesis, hydrogen metabolism, and components of the electron transport chain. (**D**) Genes involved in Fe-S cluster biogenesis, purine and pyrimidine biosynthesis, amino acid biosynthesis, and cell division, (**E**) Genes encoding ribosomal proteins. B1, B2, and B3 denote the three biological replicates of the bile group, whereas C1, C2, and C3 denote the three biological replicates of the control group.

Genes relating to surface structure modification and glycosylation, such as *pglC*, *pseB*, *pseC*, and motility-associated glycosyltransferases (I5Q61_RS00460), were upregulated in bile. Motility genes showed differential regulation; components of the flagellar motor and assembly machinery, such as *fliG*, *fliH*, *fliY*, *flgH*, *flgI*, and *flgE*, were upregulated, while structural and filament-associated genes, including *flaA*, *flaG*, *fliD*, *fliS*, and *flgC*, were downregulated. The chemotaxis gene *cheV* was also downregulated, indicating a potential suppression of environmental sensing and directional motility (Figure 2A). Upregulation of *murF* and *lpoB* (peptidoglycan biosynthesis), and *kdsB* (lipooligosaccharide core biosynthesis) reflects a strengthening of the cell envelope. Expression of an *ompA* family outer membrane protein (I5Q61_RS03650) was upregulated in bile-exposed samples. Notably, the genes involved in the CmeABC multidrug efflux system, *cmeA* and *cmeC*, as well as ABC transporters *papP* and *papQ***,** were significantly upregulated, indicating activation of defense mechanisms against bile and other stressors (Figure 2B).

Genes associated with energy metabolism were broadly induced. These included *dxs* (isoprenoid biosynthesis), *napA*, *napG*, and *napH* (components of the periplasmic nitrate reductase complex involved in anaerobic nitrate respiration and iron-sulfur electron transfer), *moaA* and *modA* (molybdenum cofactor biosynthesis), and *hydC*, *hypB*, *hypC*, and *hypE* (hydrogen metabolism genes). Components of the electron transport chain, including *cycS*, *ccoN*, *ccoO*, and NADH dehydrogenase subunits, *nuoD*, *nuoH*, *nuoI*, *nuoK*, and *nuoL*, were also upregulated in bile-exposed samples. Conversely, genes involved in several key cellular pathways, including stress response genes (*groES*, *trxA*, *tpx*, *sodB*, *ffh*, *selD*, *surE*, and *perR*), membrane transporters (*exbB* and *tonB*), and iron-sulfur cluster biogenesis genes (*iscU* and *nifS*) were downregulated. Nucleotide biosynthesis genes (*pyrH* and *gmk*), branched-chain and aromatic amino acid biosynthesis genes (*leuA*, *leuB*, and *aroQ*) were also repressed. There was a general downregulation of transcriptional and translational machinery, with numerous ribosomal protein genes (*rplA*, *rplS*, *rplT*, *rplU*, *rpsB*, *rpsF*, *rpsJ*, *rpsM*, *rpsO*, *rpsR*, *rpsU*, *rpmA*, *rpmB*, *rpmH*, and *rpmI*). In addition, DNA repair (*recA* and *xseA*) and cell division genes *(pal* and *ftsY*) were also downregulated (Figure 2C,D). Additionally, a significant number of the highly upregulated or downregulated genes encoded hypothetical proteins whose functions have not yet been ascertained.

### 3.3. Transcriptomic Adaptation of C. hepaticus During In Vitro LMH Infection

**Figure 3:**
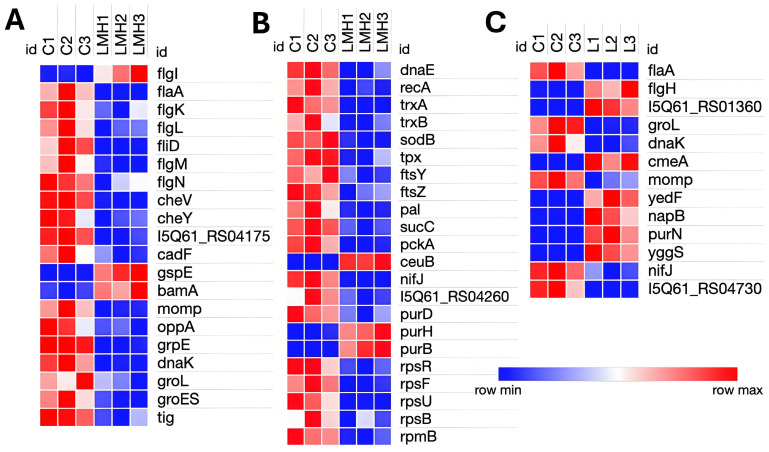
Heatmap of differentially expressed genes in *C. hepaticus*: LMH-infected and liver-infected samples vs. controls (bacteria grown in Brucella broth under standard microaerophilic cultures). (**A**) Genes involved in motility, chemotaxis, adhesion, outer membrane protein assembly, Type II secretion system, and protein folding/stress response in LMH-infected cells. (**B**) Genes associated with DNA repair, cell division, core metabolic enzymes, iron transport, purine biosynthesis, and ribosomal proteins in LMH-infected cells. (**C**) Genes differentially expressed in liver-isolated *C. hepaticus*, including those related to motility, stress response chaperones, multidrug efflux systems, outer membrane proteins, sulfur and nitrate metabolism, purine biosynthesis, and electron transport. LMH1, LMH2, and LMH3 represent the three biological replicates of the LMH-infected group; L1, L2, and L3 represent the liver-infected group; and C1, C2, and C3 represent the control groups.

Transcriptomic analysis of *C. hepaticus*-infected LMH identified 125 DEGs, including 29 upregulated and 96 downregulated genes. Motility genes exhibited opposing patterns of expression, with *flgI* (flagellar basal body) upregulated, and structural elements (*flaA*, *flgK*, *flgL*, and *fliD*) and regulators *(flgM* and *flgN*) were downregulated. Chemotaxis-related genes (*cheV*, *cheY*, and methyl-accepting chemotaxis protein, *I5Q61_04175*) were downregulated. Among the secretion system genes, *gspE* (type II secretion system protein) and *bamA* (outer membrane assembly) were upregulated, whereas *momp* (major outer membrane protein) was downregulated. The *ceuB* iron transport gene was upregulated, but *nifJ* (electron transport) and the iron-sulfur cluster assembly gene I5Q61_RS04260 were downregulated. Protein folding chaperone genes, such as *grpE*, *dnaK*, *groES*, *groEL*, and *tig*, were markedly downregulated. The purine biosynthesis genes had mixed expression levels; notably, *purH* and *purB* were upregulated, whereas *purD* was downregulated. Several genes involved in basic cellular processes, including ribosomal proteins (*rpsR*, *rpsF*, *rpsU*, *rpsB*, and *rpmB*), DNA repair *(dnaE* and *recA*), and the ABC transporter (*oppA*), were downregulated as well. Furthermore, antioxidant defense genes (*sodB*, *tpx*, *trxA*, and *trxB*), essential metabolic enzymes *(sucC* and *pckA*), the adhesion gene *cadF*, and the division genes *ftsZ*, *ftsY*, and *pal* were downregulated (Figure 3A,B).

### 3.4. Transcriptomic Analysis of C. hepaticus Isolated from Infected Livers

Transcriptomic analysis of *C. hepaticus* isolated from liver tissue identified 26 DEGs, of which 9 were upregulated and 17 were downregulated. Among the motility-associated genes, *flaA* was downregulated, while *flgH* exhibited upregulation. Similar to the bile environment, liver-infected *C. hepaticus* also showed upregulation of the gene involved in the CmeABC multidrug efflux system, *cmeA*, alongside a glycosyltransferase protein 2 gene (I5Q61_RS01360). Evidence of metabolic adaptation was detected by the upregulation of *yedF* (sulfur metabolism) and *napB* (a component of the nitrate reductase complex), which facilitates anaerobic respiration. In the purine biosynthesis pathway, *purN* was upregulated. In contrast, the outer membrane porin *momp* and stress response chaperones *dnaK* and *groL* were downregulated, along with the electron transport gene *nifJ*. Genes related to intracellular adaptation, such as *yggS*, which plays a role in PLP homeostasis, were upregulated. Furthermore, a DUF2920 family protein (I5Q61_RS04730) was downregulated (Figure 3C). Some of the genes that were highly upregulated were annotated as hypothetical proteins. Collectively, these results indicate that *C. hepaticus* adopts a metabolically poised and low-activity condition within liver tissue, thereby facilitating immune evasion and persistence, which may contribute to the pathogenesis of SLD. 

### 3.5. Confirmation of RNA-Seq Profiles by RT-qPCR

**Figure 4:**
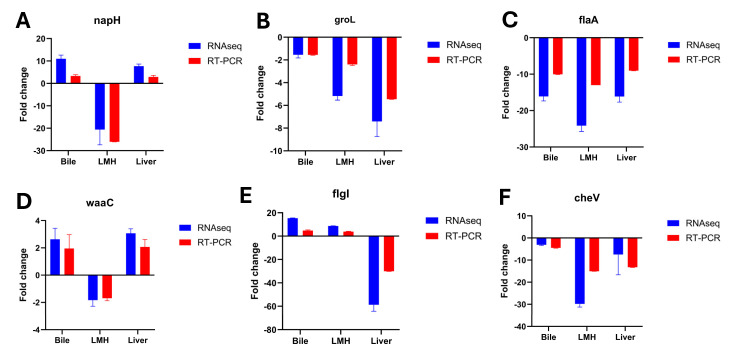
Validation of RNA-seq data by RT-qPCR. Relative expression levels of six representative *C. hepaticus* genes, *napH* (**A**), *groL* (**B**), *flaA* (**C**), *waaC* (**D**), *flgI* (**E**), and *cheV* (**F**) in bile, LMH, and liver samples were compared between RNA-seq (blue bars) and RT-qPCR (red bars). RT-qPCR confirmed the expression trends identified by RNA-seq, with both methods showing consistent upregulation or downregulation across the three experimental conditions. Error bars represent the standard deviation of three biological replicates.

To verify the transcriptional profiles identified by RNA-seq, six representative genes (*napH*, *groL*, *flaA*, *waaC*, *flgI*, and *cheV*) were analyzed by RT-qPCR. As shown in Figure 4, the expression trends observed by RT-qPCR were consistent with the RNA-seq data across bile, LMH, and liver samples, with both methods showing similar upregulation or downregulation trends. These results confirmed the reliability of the RNA-seq expression patterns.

## 4. Discussion

Our transcriptomic analysis of *C. hepaticus*, under three distinct growth conditions, namely in vitro exposure to chicken bile, infection of LMH, and isolation from naturally infected chicken livers, revealed that *C. hepaticus* extensively reprograms its gene expression, enabling it to survive under diverse environmental conditions and stresses. Although each condition prompted unique responses, several general trends were also observed.

### 4.1. Adaptation to Bile Exposure

Bile, rich in deoxycholate, is a hostile and detergent-rich environment that can damage the membranes and create oxidative stress [25]. In response to this harsh environment, *C. hepaticus* undergoes extensive transcriptomic reprogramming to fortify its cell envelope, modulate motility, alter metabolism, and activate its defenses [12]. We observed a significant upregulation of genes involved in surface structure modification and cell envelope biogenesis in the bile. For example, glycosylation-related genes (*pglC, pseB, pseC*, and motility-associated glycosyltransferase) were induced, suggesting active remodeling of the surface architecture. Consistently, *C. hepaticus* upregulated peptidoglycan biosynthesis genes (*murF*) and periplasmic peptidoglycan assembly factors *(lpoB*), along with an LOS core biosynthesis gene *(kdsB*), indicating that it reinforced its cell wall and outer membrane under bile exposure. An outer membrane protein of the OmpA family (OmpA) also showed high expression, potentially adjusting membrane permeability. CmeABC is a well-known Resistance-Nodulation-Division (RND) family efflux system in *Campylobacter* that is essential for bile resistance and intestinal colonization [26]. The upregulation of *cmeA* and *cmeC* by *C. hepaticus* in bile likely represents a rapid adaptive response to expel toxic bile components, thereby enhancing bacterial survival. These transcriptional changes indicate that the bacterium reinforces and remodels its surface membrane to combat the toxic effects of bile. This observation strongly agrees with the findings in *C. jejuni*, where LOS structure activity alteration enhances bile resistance [27]. Similarly, the *papP/Q* transporter (involved in glutamine uptake) has been implicated in stress tolerance and virulence of *C. jejuni.* The strong upregulation of *papP/Q* genes in *C. hepaticus* under bile exposure suggests that this pathogen likewise activates nutrient-scavenging and stress-protective transport systems to withstand bile-induced stress [12,27].

Bile exposure triggered a complex change in *C. hepaticus* motility gene expression. Interestingly, several genes encoding the flagellar motor and assembly apparatus (*fliG*, *fliH*, *fliY*, *flgH*, *flgI*, and *flgE*) were upregulated, whereas the major structural components of the flagellum (*flaA*, *flaG*, *fliD*, *flgC*, and *fliS*) were downregulated. The chemotaxis signaling gene *cheV* was also strongly repressed. This pattern suggests *C. hepaticus* might be fine-tuning its motility by maintaining or strengthening the flagellar motor function while limiting production of the exposed filament components. By downregulating *flaA* and related structural proteins, the bacterium may restrict flagellar assembly to conserve energy or avoid immune recognition, while preserving the machinery required for motility. Flagellin is a major antigen, and many pathogens downregulate flagellar expression in host environments to evade immune detection [12]. Indeed, a previous in vivo study found that numerous flagella and chemotaxis genes were downregulated in *C. hepaticus* recovered from the bile of infected chickens [12]. Our in vitro findings mirror this defensive strategy. In contrast, *C. jejuni* has been shown to transiently increase *flaA* expression upon bile exposure [27], presumably to enhance motility for initial stages of gut colonization. The divergence observed in *C. hepaticus* may reflect a different survival strategy, whereby early bile exposure leads to reduced flagellar filament production to minimize host immune stimulation, as our data indicated, aligning with the long-term suppression of flagella observed in vivo [12]. We speculate that *C. hepaticus* briefly relies on its existing motility organelle to navigate through bile, but quickly downregulates flagellin and chemotaxis receptors (e.g., *cheV*) once within the biliary environment, adopting a more sessile or stealthy state. This nuanced motility response likely enables the bacterium to balance the need for dissemination with the need to persist undetected in the gallbladder or bile ducts.

Bile exposure triggered significant changes in *C. hepaticus* energy metabolism, marked by the upregulation of genes involved in anaerobic and alternative respiration. Notably, the periplasmic nitrate reductase operon genes (*napA*, *napG*, and *napH*) were strongly upregulated, suggesting the use of nitrate as a terminal electron acceptor under oxygen-limited conditions in bile [12]. Key components of the cbb3-type cytochrome c oxidase (*ccoN* and *ccoO*) and NADH dehydrogenase subunits (*nuoD*, *nuoH*, *nuoI*, *nuoK*, and *nuoL*) were also upregulated, indicating a reorganization of the electron transport chain to maintain energy production. Additionally, in our study, *C. hepaticus* elevated hydrogen metabolism genes (*hydC*, *hypB*, *hypC*, and *hypE*), which is consistent with the use of molecular hydrogen for respiration, as observed in *Campylobacter* [12] and *Helicobacter* spp. [28]. The upregulation of (*dxs, moaA* and *modA*) further supports metabolic adaptation to sustain membrane function and redox balance [29,30]. Collectively, these responses suggest that *C. hepaticus* shifts toward anaerobic respiration and energy conservation to survive in the bile-rich, microaerobic environment.

Conversely, downregulation of genes involved in growth and housekeeping functions, such as multiple ribosomal proteins (*rplA*, *rplS*, *rpsB*, and *rpsM*), DNA replication and cell division (*ftsY*, *pal*, *recA*, and *xseA*), nucleotide biosynthesis (*pyrH*, *gmk*), and amino acids biosynthesis (*leuA*, *leuB*, and *aroQ*), along with several classical oxidative stress response genes (*groES*, *trxA*, *tpx*, *sodB*, and *perR*), suggests that *C. hepaticus* adopts a semi-dormant, energy-conserving state by minimizing metabolic output and potentially reducing expression of immunogenic proteins. This semi-dormant state is specifically referred to as a viable but nonculturable (VBNC) state [31]. These similar transcriptional profiles observed in *C. hepaticus* recovered from chicken bile indicate a quiescent adaptation strategy [12]. The suppressed stress response and heat-shock genes may reflect either a stabilized post-stress state or an unconventional survival mechanism. Rather than arresting growth entirely, *C. hepaticus* appears to selectively divert resources toward envelope fortification, transporter activation, and metabolic remodeling. Notably, many strongly regulated genes remain hypothetical, hinting at novel, uncharacterized factors involved in adaptation. Overall, this transcriptomic profile reflects a bacterium in survival mode, modulating its physiology for persistence in the bile-rich hepatic niche and contributing to its role in SLD.

### 4.2. Adaptation Within LMH 

Within LMH, *C. hepaticus* exhibited broad-scale gene downregulation, reflecting a transition to a low-activity state that is probably intended to avoid host defenses and adapt to the hepatocellular microenvironment intracellular niche. Importantly, the LMH cell line was selected because it provides a well-characterized in vitro hepatocyte model that closely mimics the intracellular environment of chicken liver cells, enabling us to examine *C. hepaticus* adaptations during host cell invasion and persistence. This rationale is supported by previous in vitro infection experiments, which showed that *C. hepaticus* was strongly invasive in LMH and had much higher internalization compared to *C. jejuni*, *C. lari*, and *C. upsaliensis*, with a high bacterial population localized intracellularly by 5 h post-infection [8]. Thus, in our 12 h post-infection model, we reasonably assumed that most *C. hepaticus* cells had been internalized and were actively adapting to the intracellular environment of hepatocytes.

Remarkably, motility and chemotaxis genes, such as central flagellar constituents (*flaA*, *flgK/L*, and *fliD*) and regulators *(flgM* and *flgN*), were strongly downregulated. As mentioned above under ‘Adaptation to Bile Exposure’, flagellin is a potent immune stimulus through TLR5; repression of flagellar expression may help the bacteria evade host immune detection [32,33]. This mirrors the mechanisms observed for *C. jejuni*, which downregulates flagella upon long-term colonization of the host to minimize immune activation [12,34,35]. Likewise, chemotaxis genes (*cheY*, *cheV*, and methyl-accepting receptors or transducer-like protein, I5Q61_04175) were also repressed, indicating a decreased requirement for movement within the static intracellular habitat and a transition toward a sessile, adapted state. In contrast to the broad downregulation of motility and chemotaxis genes, *C. hepaticus* upregulated some secretion and membrane-related genes, implying specific adaptations for intracellular survival. For example, *gspE*, a component of the type II secretion system, was induced, which may facilitate the secretion of effectors involved in modulating host functions or obtaining nutrients [36]. Additionally, *bamA*, which participates in outer membrane protein assembly, was upregulated, whereas *momp* was downregulated, suggesting membrane remodeling, perhaps to decrease permeability or conceal surface antigens. Furthermore, *cadF*, an important adhesin necessary for initial host cell binding and invasion, was downregulated in intracellular *C. hepaticus*, likely to reflect both a reduced need for adhesion post-invasion and a strategy to further evade immune recognition.

Downregulation of growth-related genes and protein synthesis implies that *C. hepaticus* enters a reduced or dormant phenotype within host cells [37]. Decreased expression of ribosomal proteins *(rpsR*, *rpsF*, *rpsU*, *rpsB*, and *rpmB*), replication/repair genes (*dnaE* and *recA*), and the cell division genes *ftsZ*, *ftsY*, and *pal* are indicative of a general inhibition of growth and cell division machinery. This transcriptional profile is consistent with the hypothesis that *C. hepaticus* does not actively proliferate within LMH but instead adopts a low-activity, intracellular state [5]. Interestingly, despite entering a slower growth phase, *C. hepaticus* downregulated many chaperone and stress response genes in LMH, including *grpE*, *dnaK*, *groES*, *groEL*, and *tig*. This suggests that the intracellular environment may be less stressful than external conditions, like growth in bile or bacteriologic media under microaerophilic conditions. Suppression of highly immunogenic proteins, such as *groEL* and *dnaK*, may further help the bacterium evade immune detection. Overall, *C. hepaticus* appears to adapt to the intracellular niche without triggering a classical stress response.

Metabolic changes observed in LMH-infected *C. hepaticus* suggest a shift toward energy conservation and nutrient scavenging. Selective upregulation (*purH*, and *purB*) and downregulation (*purD*) of genes involved in purine metabolism point to partial reliance on host-derived nucleotides, allowing the bacterium to bypass the energy-intensive early steps of de novo purine synthesis. Concurrently, downregulation of central carbon metabolism genes (*sucC*, and *pckA*) indicates reduced glycolytic and TCA activity, possibly due to dependence on amino acids or fatty acids available within the nutrient-rich LMH intracellular environment. Similarly, the downregulation of antioxidant enzymes like *sodB*, *tpx*, and *trxA/B* suggests that the intracellular environment of hepatocytes does not subject the bacterium to high levels of reactive oxygen species (ROS), in contrast to phagocytic immune cells that actively generate oxidative bursts [38]. This apparent lack of oxidative stress in LMH might enable *C. hepaticus* to conserve energy by downregulating ROS-detoxifying enzymes. Additionally, the upregulation of *ceuB* in LMH environment showed that *C. hepaticus* can sense and respond to iron limitation within host cells. *CeuB*, a component of the siderophore-mediated iron uptake system in *Campylobacter*, imports ferric iron. Given that the free iron is tightly sequestered by host nutritional immunity mechanisms, the upregulation of *ceuB* likely reflects a bacterial strategy to maintain iron homeostasis and support essential metabolic processes under iron-restricted intracellular conditions by increasing iron uptake [39,40].

### 4.3. In Vivo Adaptation in the Liver

The *C. hepaticus* gene expression profile in infected liver tissue shares many similarities with that observed in the bile exposure and LMH infection models. In addition, liver tissue samples from naturally infected chickens were included to capture the in vivo hepatic environment, reflecting complex host–pathogen interactions, immune pressure, and nutrient availability during actual disease progression. The liver, the primary site of SLD lesions, is a nutrient-rich environment but also exposes the bacterium to the immune defense of the host and antimicrobials like bile salts and complement proteins derived via the bloodstream and bile ducts [41]. As in the bile and LMH intracellular niches, *C. hepaticus* in the liver downregulated prominent motility gene *flaA*, which supports the hypothesis that diminished motility facilitates immune evasion within the target organ. This finding aligns with the observations in *C. jejuni*, where long-term colonization of the host is often associated with downregulation of flagellar and chemotaxis genes, likely to avoid detection by host immune cells [12,34,42]. Notably, despite the overall suppression of flagellar genes, *flgH*, which encodes the L-ring of the flagellar basal body, was upregulated. Given its role in anchoring the flagellum to the outer membrane, this solitary upregulation of *flgH* might reflect a need to stabilize the flagellar base or strengthen the outer membrane during stress conditions [43]. Alternatively, *flgH* expression could be a result of operon-level transcriptional control or an indication of host-driven signaling subtleties [44].

The key adaptation of *C. hepaticus* in the liver is its change in respiratory metabolism. Upregulation of *napB* suggests reliance on nitrate as an alternative electron acceptor. This shift may confer an energetic advantage or reflect greater availability of nitrate/nitrite due to hepatic inflammation. This mirrors the findings that *C. jejuni* readily switches to nitrate/nitrite respiration under in vivo bile conditions, and the upregulation of *nap* genes has been documented during bile exposure [12]. This is consistent with the observed upregulation of the nitrate reductase gene (*napA*) in our in vitro bile experiment. By prioritizing nitrate respiration, *C. hepaticus* can produce energy in low-oxygen environments and may also reduce harmful reactive nitrogen species from the host through baseline *nrf* gene activity, which provides two key advantages for survival in the host tissues. Beyond respiration, several other metabolic genes of *C. hepaticus* were also differentially expressed in the liver. For example, *yedF*, a gene potentially engaged in sulfur metabolism, was upregulated. The liver and gallbladder are rich in sulfur-containing compounds (e.g., taurine-conjugated bile acids) [45], so elevated *yedF* expression may enhance the bacterium’s ability to utilize or detoxify sulfur compounds available in this environment. Additionally, *yggS*, which is involved in the preservation of pyridoxal phosphate (PLP), was also induced. As PLP is vital for amino acid metabolism and oxidative stress protection, elevated *yggS* likely supports PLP-dependent enzyme activities during infection, indicating another level of fine-tuning of bacterial metabolism in the host [46]. Changes in nucleotide biosynthesis further suggest metabolic reprogramming of *C. hepaticus* in the liver. Upregulation of *purN* suggests a possible shift toward purine salvage or late-stage synthesis, likely enabled by the availability of host-derived precursors such as glycine or formate. This may also reflect feedback inhibition in response to abundant purines released by lysed host cells.

An important virulence gene, *cmeA*, encodes the periplasmic adaptor protein of the CmeABC multidrug efflux pump, which plays a key role in bile salt resistance in *Campylobacter* [18]. Similar to our bile exposure experiment, *cmeA* overexpression in the liver suggests that *C. hepaticus* activates the CmeABC efflux pump to detoxify bile salts and other toxic compounds present in the hepatic environment. This mechanism, also used by *C. jejuni*, contributes to antimicrobial resistance and allows bacterial survival within host tissues [27,47]. In contrast, the major porin gene *momp* was downregulated in liver-derived *C. hepaticus*, likely reducing outer membrane permeability thereby limiting the entry of bile salts and host complement molecules through porin channels. While this may restrict nutrient intake, it enhances defense in the hostile liver environment. This pattern of porin downregulation coupled with efflux pump activation reflects a well-known stress response in *C. jejuni* and other Gram-negative bacteria under bile exposure [18,48]. The similar transcriptomic changes observed in the *C. hepaticus* LMH infection model (upregulation of *gspE* and *bamA*, and downregulation of *momp*) further support the notion that outer membrane remodeling represents a consistent survival strategy within host cells and tissues. Along with porin changes, a glycosyltransferase gene (I5Q61_RS01360) was strongly upregulated in liver-isolated *C. hepaticus*, indicating potential modifications to surface structures such as the capsule or LOS. In *Campylobacter*, surface glycosylation of LOS, capsule, and flagellin is a known strategy for immune evasion and host mimicry [49]. Both in vitro LMH and in vivo liver isolates, *C. hepaticus* downregulated classic heat-shock or general stress response chaperone genes *groL* and *dnaK* (Hsp70). These molecular chaperones typically help refold misfolded proteins under stress but are also highly immunogenic and can trigger strong host immune responses when exposed to the cell surface or released during infection [50]. Repression of these genes in the liver points to two possible explanations: (1) the bacterium resides in a relatively stable intracellular/bile environment that does not elicit a stress response, or (2) it intentionally suppresses these immunostimulatory proteins to minimize immune detection. This strategy contrasts sharply with many acute infections where bacteria upregulate stress proteins and inadvertently provoke robust immunity [50,51]. Collectively, these transcriptomic results suggest that *C. hepaticus* adopts a metabolically poised, dormant state within liver tissue. Through downregulation of strong immunogenic proteins, *C. hepaticus* can evade immediate immune clearance to form chronic infection foci. However, constant metabolic activities, such as ammonia production via nitrite reduction, can provoke local inflammation and hepatocellular injury. This balance between immune evasion and sustained low-level stimulation is likely a key factor driving the formation of the characteristic necrotic lesions observed in SLD.

The number of differentially expressed genes varied across the bile, LMH, and liver niches. Bile exposure triggered the highest number of gene expression changes, consistent with the bacterium’s need to counteract detergent-rich, oxidative, and nutrient-limited conditions through envelope remodeling, efflux activity, and metabolic reorganization. In contrast, LMH infection was characterized by widespread downregulation of genes, indicative of a low-metabolic, immune-evasive state within the intracellular hepatocyte environment. Liver tissue, representing the complex in vivo milieu, showed an intermediate number of differentially expressed genes, likely reflecting simultaneous exposure to host immune defenses, nutrient limitations, and bile-derived stress. These observations suggest that *C. hepaticus* employs distinct transcriptional strategies depending on the niche, balancing active stress responses in bile with stealth and persistence mechanisms in host-associated environments.

## 5. Conclusions

This study highlights dynamic translational adaptations of *C. hepaticus* across distinct host-relevant environments, such as chicken bile, hepatocytes (LMH), and infected liver tissue, demonstrating dynamic niche-specific gene expression adaptations. In bile, *C. hepaticus* exists in a metabolically active, semi-dormant state, with remodeling of the cell envelope, activation of efflux, and induction of alternative respiratory pathways to resist detergent stress and oxygen restriction. In contrast, in hepatocytes and infected livers, the bacterium assumes a low-active, dormant, immune-evasive phenotype, where most motility, protein synthesis, and immunogenic stress protein genes are repressed, but key metabolic processes, such as nitrate respiration, sulfur utilization, and pyridoxal phosphate homeostasis, are preserved. These results underscore the two-phase infection strategy employed by *C. hepaticus*: an initial phase of active colonization and resistance during initial biliary transit, followed by a phase of intracellular persistence in host tissues mediated by metabolic reprogramming and immune evasion. Targeting these phase-specific processes may provide effective strategies for controlling SLD. For example, vaccines against outer membrane iron transporters or flagellum components may impair bacterial colonization and fitness, whereas inhibitors of hydrogenase or nitrite reductase may selectively weaken *C. hepaticus* without disrupting the commensal gut microbiota. Additional functional studies involving gene knockouts or knockdowns will be necessary to validate these targets and further elucidate the molecular mechanisms underlying *C. hepaticus* virulence. Overall, this transcriptomic analysis of *C. hepaticus* in multiple host-relevant niches enhances our knowledge of *C. hepaticus* pathogenesis and identifies promising targets for intervention.

## Data Availability

The original data presented in the study are openly available in the Gene Expression Omnibus (GEO) database in NCBI under the accession number GSE305414.

## Supplementary Materials

The following supporting information can be downloaded at: https://www.mdpi.com/article/10.3390/pathogens14101048/s1, File S1: Overall differential gene expression profile data in bile, LMH, and infected liver using *C. hepaticus* UF2019SK1 as reference genome; File S2: Overall differential gene expression profile data in bile, LMH, and infected liver using *C. hepaticus* HV10 as reference genome; Figure S1: Heat map of all differentially expressed genes of *C. hepaticus* in bile; Figure S2: Heat map of all differentially expressed genes of *C. hepaticus* in LMH; Figure S3: Heat map of all differentially expressed genes of *C. hepaticus* in the infected liver; Table S1: Primers used for RT-qPCR analysis.

## Abbreviations

The following abbreviations are used in this manuscript:

SLD	Spotty liver disease
LMH	Chicken liver hepatocellular carcinoma epithelial cells
FBS	Fetal bovine serum
FDR	false discovery rate
DEG	Differentially expressed gene
RIN	RNA integrity number
FC	Fold change
LOS	Lipooligosaccharide
VBNC	Viable but non-culturable
