# Supplementary material for: Campylobacter hepaticus Transcriptomics Identified Genes Involved in Spotty Liver Disease (SLD) Pathogenesis
Source: Pathogens. 2025 Oct 17;14(10):1048. doi: 10.3390/pathogens14101048 (PMC12567157; doi:10.3390/pathogens14101048)
Supplement: Supplementary file 1 [file pathogens-14-01048-s001.zip › Supplementary Figures.pdf]

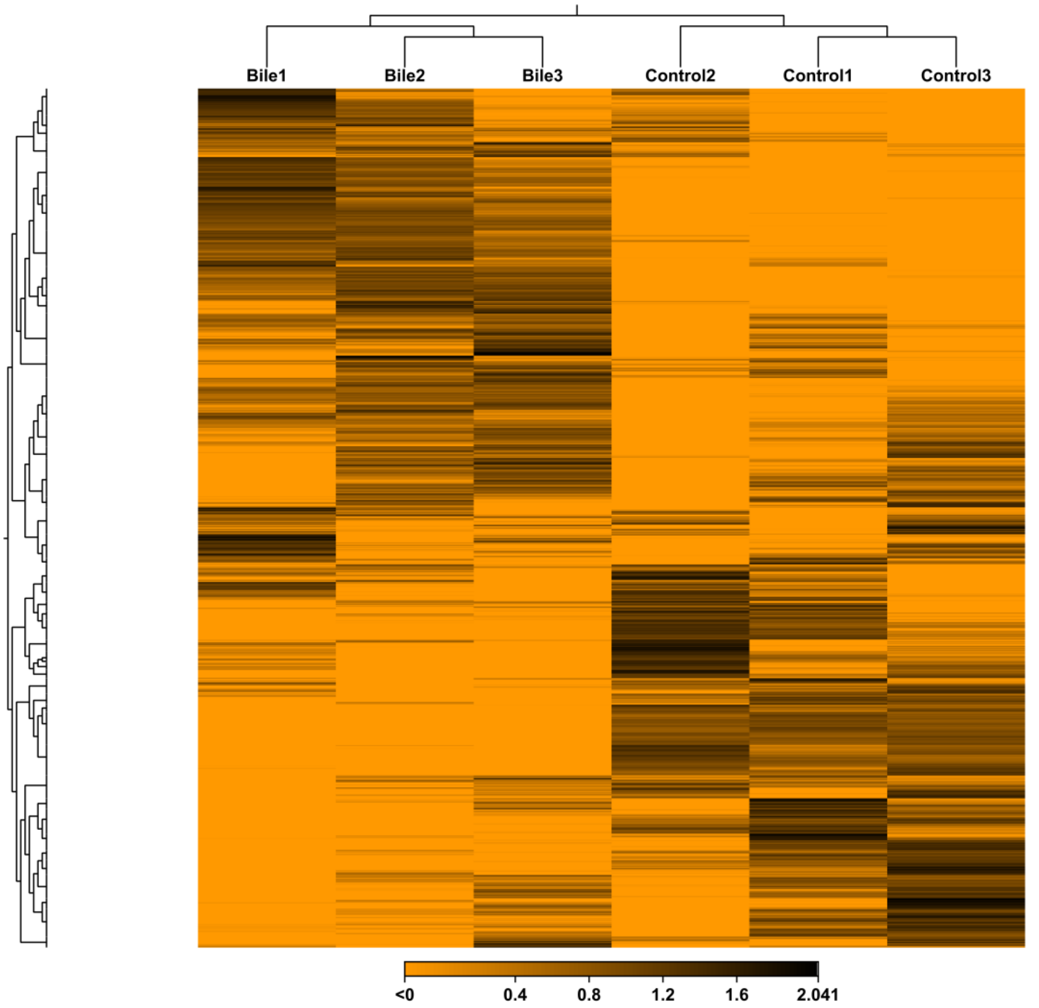

Figure S1. Heat map of all differentially expressed genes of *C. hepaticus* in bile.

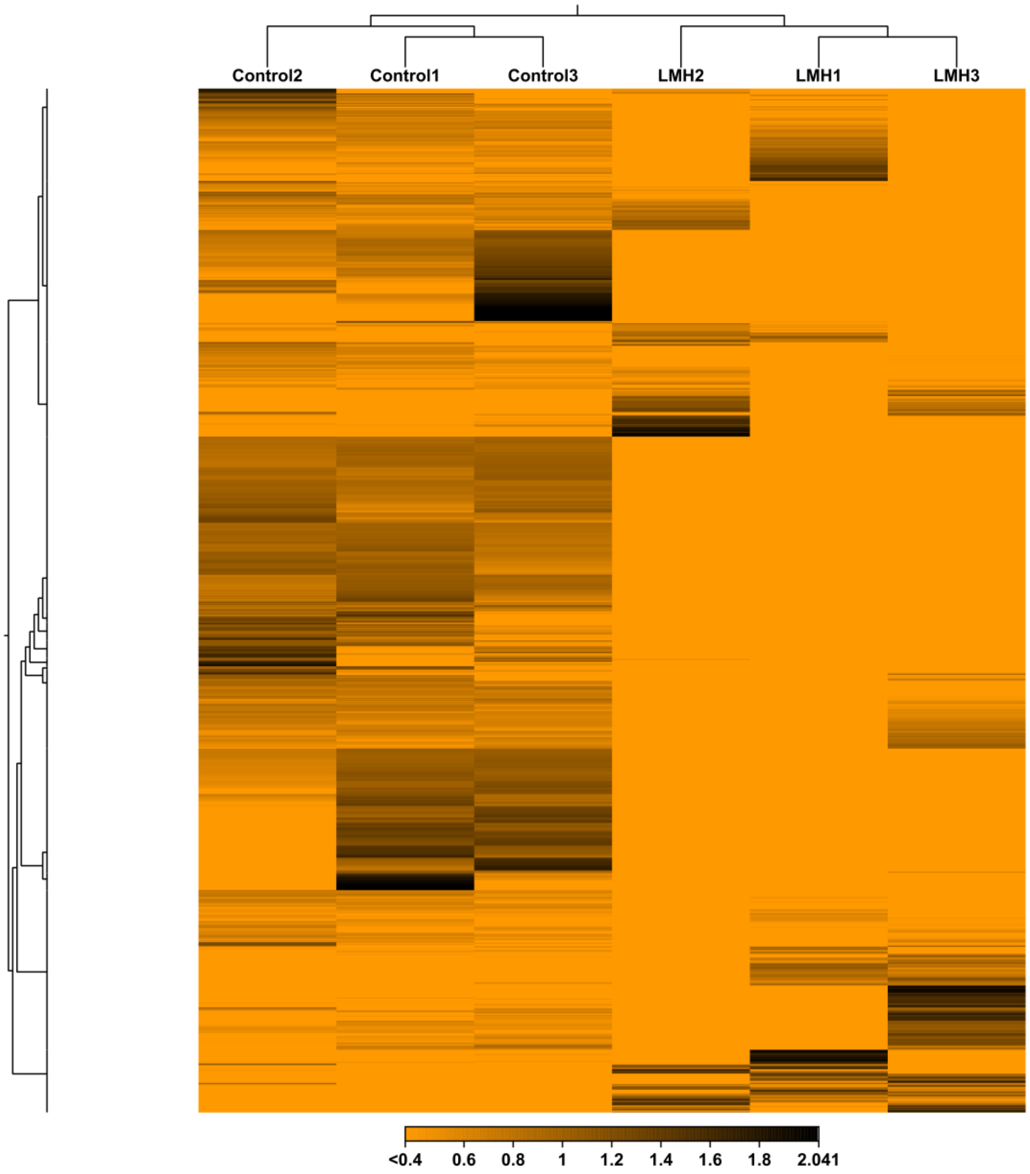

Figure S2. Heat map of all differentially expressed genes of *C. hepaticus* in LMH cells.

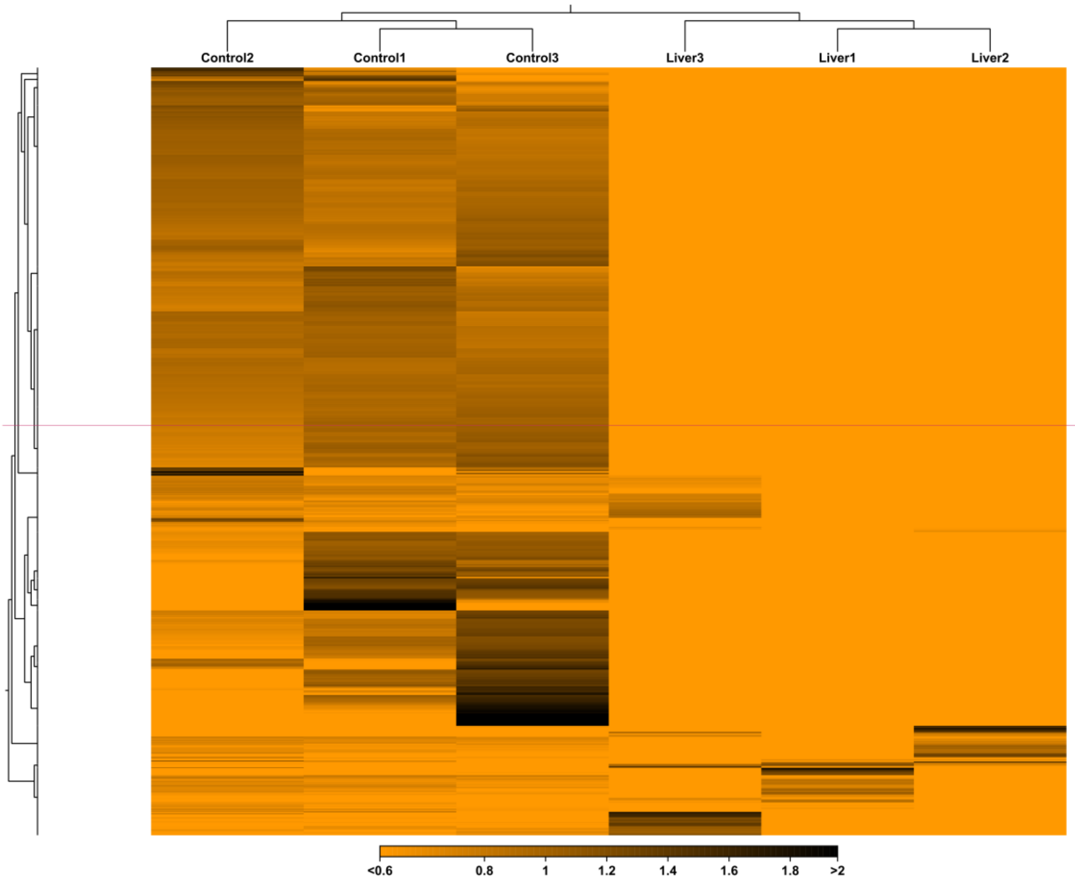

Figure S3. Heat map of all differentially expressed genes of *C. hepaticus* in the infected liver.
