# Supplementary material for: Campylobacter hepaticus Transcriptomics Identified Genes Involved in Spotty Liver Disease (SLD) Pathogenesis
Source: Pathogens. 2025 Oct 17;14(10):1048. doi: 10.3390/pathogens14101048 (PMC12567157; doi:10.3390/pathogens14101048)
Supplement: Supplementary file 1 [file pathogens-14-01048-s001.zip › Supplementary Table -Primers used for qRT-PCR.pdf]

**Table S1. Primers used for qRT-PCR**

| Gene | Primer | Sequence (5′-3′) |
|---|---|---|
| rpoB | Forward | CGTCAAGCTGTACCGCTTCT |
| | Reverse | TCAACCACTCCACCACGTTT |
| napH | Forward | AGCCATAATTCACAGGGGGATT |
| | Reverse | AGTGGACAAAAATGCGAGCAA |
| groL | Forward | GGTGCAGCAACAGAAACTGAA |
| | Reverse | TGCGCCACCACCAATAACTA |
| cheV | Forward | ATCTTACAGAGCTTCCAGGCG |
| | Reverse | AGGCTCGGTAATTTGCATCCA |
| waaC | Forward | GCAAAAATTTGGCTAGCTTGGG |
| | Reverse | GGTTGAATTTGGGTGCTAAGCT |
| flaA | Forward | TAAGCGGGTCAGGTCTTTCAG |
| | Reverse | ATCCCATAGCATCCGCAACAT |
| flgI | Forward | TGACAGCCAAATTACCCGCT |
| | Reverse | AGTAAAAGCGTTCCCCCTTGT |
